# How ALD Settings May Affect the Chemical Structure of the (Zn_1–*x*_Sn_*x*_)O_*y*_/Wide-Gap (Ag,Cu)GaSe_2_ Thin-Film Solar Cell Interface

**DOI:** 10.1021/acsami.5c21162

**Published:** 2026-01-16

**Authors:** Angelika Demling, Jan Keller, Regan G. Wilks, Carl Hägglund, Marika Edoff, Marcus Bär

**Affiliations:** † Department of Interface Design, 28340 Helmholtz-Zentrum Berlin für Materialien und Energie GmbH (HZB), 12489 Berlin, Germany; ‡ Division of Solar Cell Technology, Department of Materials Science and Engineering, 8097 Uppsala University, P.O. Box Uppsala 35-751 03, Sweden; § Energy Materials In-Situ Laboratory Berlin (EMIL), HZB, 12489 Berlin, Germany; ∥ Department of X-ray Spectroscopy at Interfaces of Thin Films, Helmholtz-Institute Erlangen-Nürnberg for Renewable Energy (HI ERN), 12489 Berlin, Germany; ⊥ Department of Chemistry and Pharmacy, Friedrich-Alexander-Universität Erlangen-Nürnberg (FAU), 91058 Erlangen, Germany

**Keywords:** ACGSe, chalcopyrite, ZTO, alternative buffers, solar cells, HAXPES, ALD

## Abstract

In this work, the effect of different atomic layer deposition (ALD) settings on the interface formation between ALD-grown (Zn_1–*x*_Sn_*x*_)­O_*y*_ (ZTO) buffers with varying [Sn]/([Sn]+[Zn]) composition and wide-band gap silver-alloyed CuGaSe_2_ (ACGSe) absorbers is investigated using X-ray fluorescence, transmission electron microscopy, and synchrotron-based soft and hard X-ray photoelectron spectroscopy. For buffer layers prepared in an industry-scale ALD reactor, we discover the formation of an interlayer best described by a mixture of ZnO with a minor (<10%) amount of Ga_2_O_3_, which is not present in similar layer stacks prepared in a lab-scale R&D-type ALD reactor. In addition, we show that ZTO/ACGSe-based solar cells containing such an interlayer have significantly lower fill factors compared to their counterparts without this interlayer, suggesting the presence of an electron transport barrier at the front contact.

## Graphical abstract

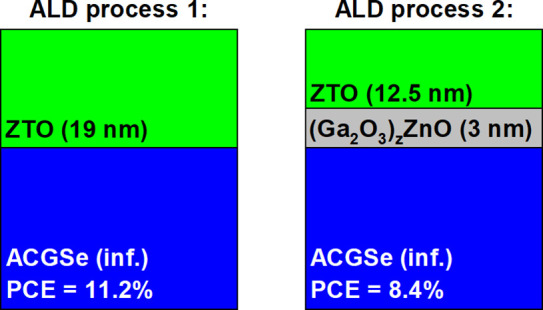

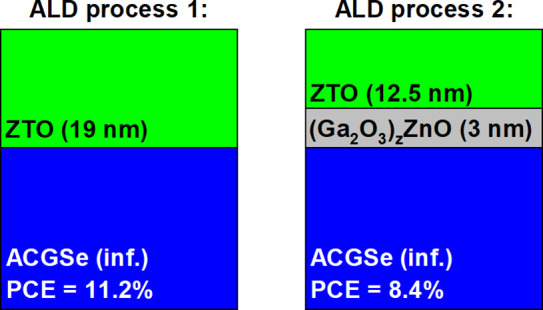

## Introduction

The chalcopyrite semiconductor CuGaSe_2_ exhibits a bandgap energy of 1.6–1.7 eV,^1–3^ which makes it a promising absorber material for top solar cells in two-junction tandem devices. Unfortunately, it suffers from Shockley–Read–Hall recombination appearing at grain boundaries^4,5^ and defect sites in the bulk^6–11^ as well as interface recombination at the buffer/absorber interface.^12–14^ Silver alloying has been shown to enlarge grain size and suppress formation of undesired, secondary phases such as Ga_2_Se_3_, to increase minority carrier diffusion lengths, and is discussed to mitigate deep defect levels.^14,15^ Additionally, silver alloying allows a reduction of process temperature during absorber deposition, inhibiting undesired gallium oxide formation at transparent back contacts necessary in tandem devices.^16,17^ The recombination losses at the buffer/absorber interface are caused by a negative conduction band offset between CuGaSe_2_ and standard CdS buffers.^18,19^ This issue is not unique for CuGaSe_2_ but applies to wide-band-gap chalcopyrite semiconductors in combination with CdS buffers in general.^3,14,20–23^ (Zn_1–*x*_Sn_*x*_)­O_*y*_ (ZTO) grown by atomic layer deposition (ALD) offers a suitable alternative buffer for these types of absorbers due to its large band gap and electron affinity, which can be adapted by changing the Sn to metal ([Me] = [Sn] + [Zn]) ratio ([Sn]/[Me]).^24^ That is very likely the reason why the highest efficiencies of CuGaSe_2_ solar cells using a ZTO buffer exceed the ones of cells employing CdS buffers significantly (11.9% [not certified] vs 11.0% [certified]).^3,25^

However, the ALD of ZTO on chalcopyrite materials is a complex process. For example, the initial ALD-growth of ZTO is inhibited, likely due to the low sticking coefficient for the precursor diethyl zinc (DEZn) and/or water on the chalcopyrite, leading to an overestimation of layer thicknesses and large lateral thickness variations, especially for thin (a few tens of nm) ZTO layers.^26^ It is believed that the resulting nucleation delay is further exacerbated by incomplete removal of the Sn precursor ligands,^27^ while the continued ZTO growth rate depends in a nonlinear fashion on the number of Zn and Sn oxide cycles per supercycle, as this affects surface hydroxylation and ligand adsorption–desorption kinetics in a delicate manner.^28^ Unfortunately, when moving from lab-scale to industrial-scale processes, ALD parameters—such as heat stabilization or pulse times—must be adapted to the larger reactor volume, which can affect the ALD process (and thus the properties of the deposited layer) unexpectedly.

In this work, we use X-ray fluorescence (XRF), transmission electron microscopy (TEM), and synchrotron-based soft and hard X-ray photoelectron spectroscopy as complementary techniques to investigate the interface between silver-alloyed CuGaSe_2_ absorbers ((Ag,Cu)­GaSe_2_, “ACGSe”) and ZTO buffers with varying [Sn]/[Me] ratios prepared by ALD. For samples prepared in an industry-scale ALD reactor, we discover the formation of an interlayer best described by a mixture of ZnO and Ga_2_O_3_ ((Ga_2_O_3_)_*z*_ZnO), which is not present in similar layer stacks prepared in a smaller lab-scale R&D-type ALD reactor, and discuss its origin. Further, we find that solar cells containing this interlayer show significantly lower fill factors, suggesting the presence of a charge carrier transport barrier at the front contact. These findings underline the complexity of interface formation and the importance of careful evaluation of ALD settings.

## Methods

### Sample Preparation and Handling

#### Absorber Preparation

The ACGSe solar cell samples were processed at Uppsala University in the following sequence. First, a 320 nm-thick Mo back contact was sputter-deposited on top of high-strain point glass, followed by a 15 nm-thick NaF precursor layer, grown via thermal evaporation (no alkali diffusion barrier was used). Subsequently, a three-stage ([Cu]+[Ag] = group [I]-poor → [I]-rich → [I]-poor) coevaporation process was applied to grow 2 μm-thick ACGSe films at a maximum temperature of 650 °C. Silver was added in a way that the ratio of the Ag and Cu evaporation rates was kept constant at any time, and a heavy alkali postdeposition treatment was not implemented. The final absorber composition was [Ag]/([Ag]+[Cu]) = 0.40–0.43 with a very close-stoichiometric I/III (([Ag]+[Cu])/[Ga]) value of 0.94–0.97, suggesting the presence of only a very minor (if any) amount of ordered vacancy compounds at the absorber surface.^29^ Furthermore, no significant compositional depth grading was observed for the used deposition protocol.^19^

The prepared (5 cm × 5 cm) ACGSe/Mo/glass sample was cut into smaller pieces for the later planned, individual ZTO-ALD runs. Subsequently, the (now smaller) samples were stored in a high-vacuum chamber until the three respective ZTO-ALD runs were conducted. The total air exposure time was <20 min for all samples. After the buffer deposition, the samples were placed into N_2_-filled plastic bags and directly shipped for the hard- and soft X-ray photoelectron spectroscopy (HAXPES and XPS) analysis to the Helmholtz-Zentrum Berlin für Materialien und Energie GmbH (HZB).

#### Atomic Layer Deposition of the (Zn_1–*x*_Sn_*x*_)­O_*y*_ Buffer

ALD of (Zn_1–*x*_Sn_*x*_)­O_*y*_ (ZTO) was performed in two different reactors: a Picosun R200 Advanced reactor (R200), for up to 15 cm diameter samples, and an ASM Microchemistry F120 (F120) reactor for square samples up to 5 cm. Results based on the latter samples have already been published in an earlier study.^19^ In both cases, a substrate temperature of 120 °C was maintained during the deposition. Further, diethylzinc (DEZn, ≥52 wt % Zn basis) and tetrakis­(dimethylamido)­tin­(IV) (TDMASn, Sigma-Aldrich, 99.9% trace metals basis) were used as precursors for Zn and Sn, respectively, with deionized water as the counterreactant. An ALD super-cycle was defined using a number of ZnO cycles, followed by one or more SnO_*x*_ cycles. The super-cycle was always defined with the minimum number of cycles required to obtain a targeted Zn/Sn pulse ratio, so that the constituents were as mixed as possible in the grown film. Due to the different reactor volumes and flow geometries, the deposition conditions in the F120 reactor could not be transferred directly to R200. Rather, the number of pulses and Zn/Sn pulse ratio of the R200 process were adapted based on the film thickness and the elemental concentration of Sn to the total concentration of metal [Sn]/[Me], with the aim to prepare ZTO samples similar to the F120 samples. This required a higher Zn/Sn precursor pulse ratio in the R200 process, as detailed in Table 1, along with the other deposition conditions. The elemental concentrations were determined using XRF, performed on a PANalytical Epsilon 5 EDXRF spectrometer, and the film thicknesses were determined by spectroscopic ellipsometry using a Woollam RC2 tool. Note that in the following, X_XRF_ refers to the [Sn]/[Me] ratio of the prepared (Zn_1–*x*_Sn_*x*_)­O_*y*_ films determined by XRF, whereas X_PES_ denotes the [Sn]/[Me] ratio extracted from photoelectron spectroscopy (PES) data (as detailed below). Further, the XRF-derived film thickness given in Table 1 was measured for ZTO on glass. Generally, ZTO growth is inhibited on chalcopyrite surfaces, leading to about 10 nm thinner films, as shown in ref 26. Based on EDS and Rutherford backscattering spectrometry,^26^ the depth profiles of ZTO ALD grown on Cu­(In,Ga)­Se_2_ appear uniform, implying close to steady-state growth behavior after the initial delay. For our ALD ZTO on ACGSe and quartz glass, we also find that the thicknesses on ACGSe (based on TEM, see Figure 2) are consistent with a 10 nm growth delay when compared to ZTO deposited in parallel on quartz glass (Table 1, based on spectroscopic ellipsometry), suggesting a similar set of substrate surface interactions at play. We therefore believe that the ZTO growth on ACGSe is close to steady-state and linear after the initial incubation/substrate conditioning period, resulting in films with a close to uniform depth profile.

**Table 1:** ALD parameters for (Zn_1–*x*_Sn_*x*_)­O_*y*_ buffer layers resulting in similar values for the elemental [Sn]/[Me] ratio prepared in two different ALD reactors (F120, R200)^a^

reactor	X_XRF_ [Sn]/[Me] elemental ratio from XRF on bare glass	heat stabilization time (min)	pulse time Sn (ms)	pulse time Zn (ms)	pulse time H_2_O (ms)	purge time (ms)	pulse ratio Zn/Sn	number of super cycles	film thickness on quartz glass (nm)
F120 from ref 19	0.260	30	400	400	400	800	3:4	100	30.0
R200	0.188	∼90	100	100	100	see note^a^	5:1	48	23.5
R200	0.210	∼90	100	100	100	see note^a^	4:1	58	19.5
R200	0.252	∼90	100	100	100	see note^a^	3:1	60	21.0

a Purge times in the ZnO cycle are 3 s for DEZ and 4 s for H_2_O; purge times in the SnO_2_ cycle are 9.9 s for TDMASn and 9.9 s for H_2_O. This adds up to a total super-cycle time of 27.2 s.

**Figure 1:**
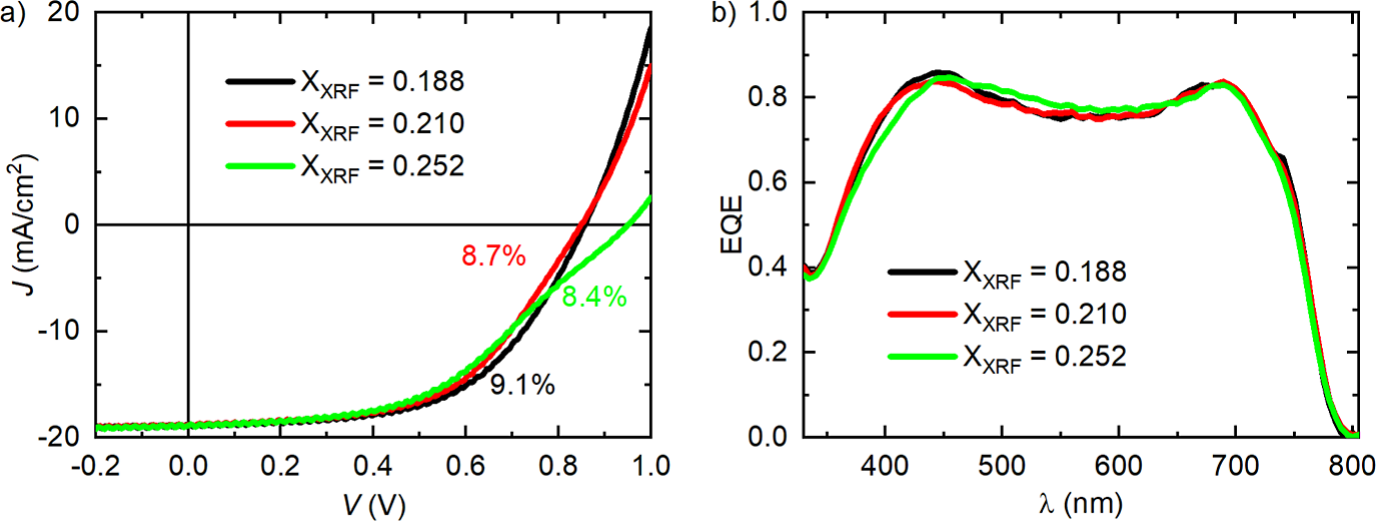
(a) *J*(*V*) characteristics of the best ZTO/ACGSe-based cells with different (Zn_1–*x*_Sn_*x*_)­O_*y*_ compositions (processed in the R200 ALD-reactor). (b) Corresponding external quantum efficiency (EQE) curves. All results after 24 h of light soaking.

**Figure 2:**
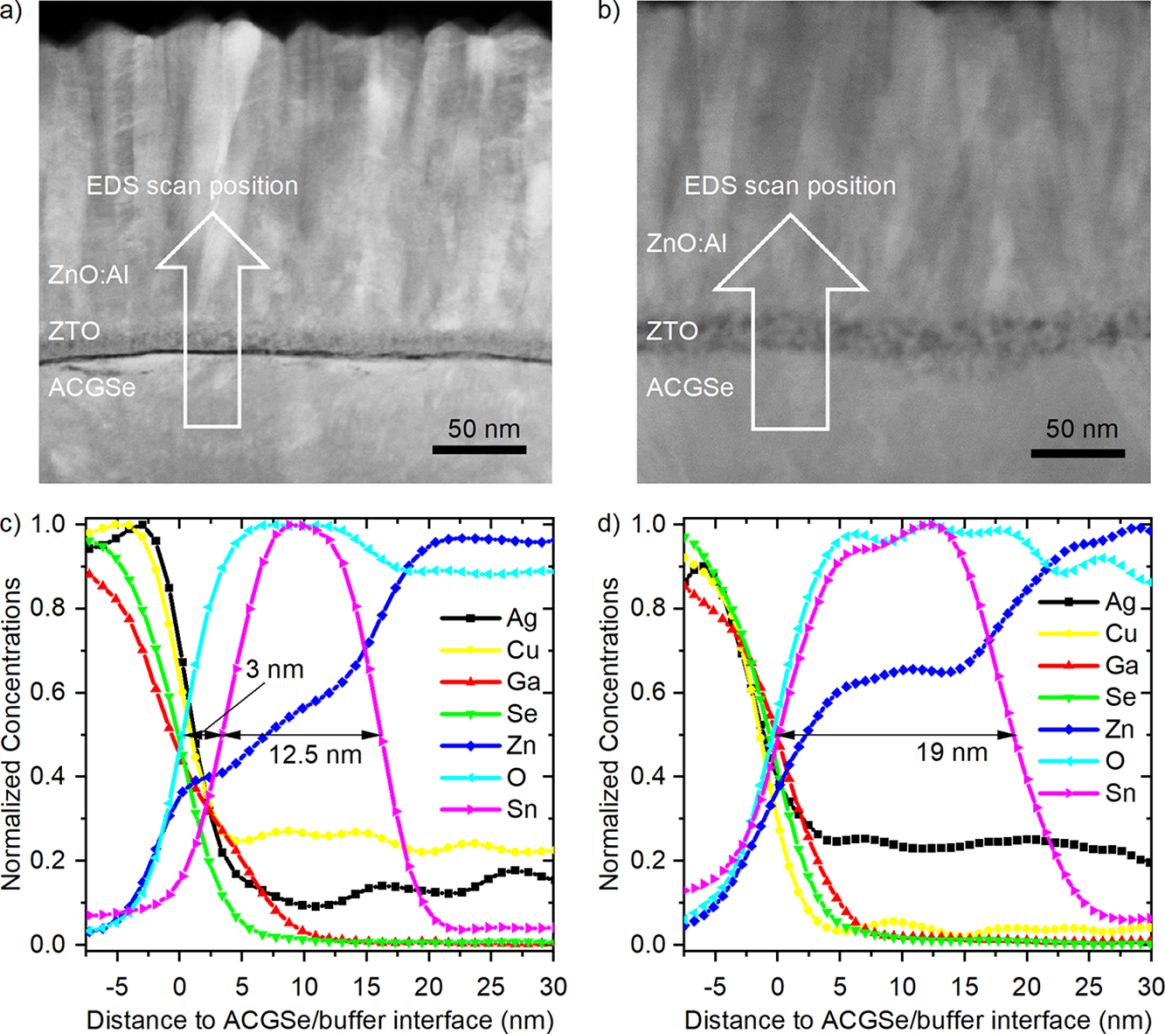
Dark-field STEM image of a complete ZnO:Al/ZTO/ACGSe solar cell stack with ZTO processed in the (a) R200 or (b) F120 ALD reactor having an X_XRF_ of 0.252 and 0.260, respectively. The arrow indicates the position, width, and direction of the EDS line scan shown as normalized atomic concentrations in (c,d). The high Cu signal outside the absorber region in (c) is an artifact of the Cu TEM grid used for the measurement of the R200-processed sample. This artifact is absent in (d) since a Ti TEM grid was used for samples with ZTO from the F120 reactor.

Note that the ZTO buffer layers with varying [Sn]/[Me] ratios from ALD reactor R200 were deposited on top of the exact same ACGSe film (i.e., from the same run). These samples were stored under vacuum and eventually sent to HZB in sealed, low-vacuum plastic bags for PES analysis (see below).

#### Solar Cell Fabrication and Device Characterization

For each ZTO composition, a small piece was reserved to complete ten solar cells by sputtering (direct-current) a 160 nm-thick ZnO:Al window layer on top of the buffer layer in an Ar atmosphere and finally defining the cell area (0.05 cm^2^) via mechanical scribing. It was required to use such small cell areas since all samples (for analysis and solar cells) stem from the same initial 5 cm × 5 cm ACGSe/Mo/glass sample, limiting the material available for solar cell fabrication. This approach allowed for the best possible (i.e., most isolated) evaluation of the effect of the [Zn]/[Sn] metal ratio in the ZTO buffer layer on the interface and solar cell device properties because differences in ACGSe surface composition are expected to be very small. The current–voltage characteristics under standard test conditions (illumination via an ELH lamp) and external quantum efficiency spectra of the solar cells were measured in home-built setups at Uppsala University.

#### Transmission Electron Microscopy

Transmission electron microscopy (TEM) cross-section lamellae of the full cell stacks were polished with a Ga-based focused ion beam model Crossbeam550 from Zeiss. The polishing always contained a low-energy 1 kV step for the final pass to lower Ga-ion implantation and reduce potential ion damage to a few nanometers in depth. Energy-dispersive X-ray spectroscopy (EDS) analyses in scanning TEM (STEM) mode were performed on a Titan Themis200 instrument at 200 kV. In the case of the sample with the ZTO layer processed with the R200 instrument, a Cu-grid was used for the analysis, leading to an artificial offset of the Cu EDS signal. For the sample with ZTO from the F120 reactor, a Ti-grid was used to avoid this artifact.

#### Synchrotron-Based X-ray Photoelectron Spectroscopy

At HZB, the packaged ACGSe samples with ZTO buffers from the R200 ALD reactor entered the UHV system through a nitrogen glovebox, where they were unpacked and mounted to avoid surface contamination.

Hard- and soft X-ray photoelectron spectroscopy (HAXPES and XPS) experiments were conducted at the SISSY-1 endstation of the Energy Materials In Situ Laboratory Berlin (EMIL) located at BESSY II. In the SISSY-1 setup, the two-color beamline of EMIL in combination with a Scienta EW 4000 hemispherical electron analyzer is used to perform photoelectron spectroscopy in the hard and soft X-ray regime under UHV conditions (base pressure <5 × 10^–9^ mbar). Hard X-rays between 2 and 10 keV are provided by the U17 DCM undulator beamline, while soft X-rays between 100 and 1500 eV are provided by the U48 DCM undulator beamline. Both beamlines are focused such that they hit the sample in SISSY-1 in the same spot. Small deviations from the nominal photon energy were corrected by referencing the Au 4f_7/2_ peak of a grounded clean Au foil to a binding energy (BE) of 84.00 eV.

The information depth of an XPS/HAXPES measurement is limited by the inelastic mean free path (IMFP) of the photoelectrons, as it is several orders of magnitude shorter than the photon attenuation length. Therefore, the photoemission intensity I emitted from a certain depth d with respect to the surface is given by the exponential function displayed in eq 1

1 $$I\left(d\right)=I_{0}\cdot e^{-d/IMFP}$$

where *I*_0_ refers to the intensity of primary electrons. That means the largest individual contribution to the measured signal always originates from the surface layer, and about 95% of the measured signal arises from within 3 × IMFP.^30^ Hence, all spectra represent an exponentially weighted average of the composition within ∼3 × IMFP. The IMFP depends on the density of the material and the kinetic energies of the photoelectrons. In this study, excitation energies (*h*ν_exc_) of 4000, 1400, and 220 eV are employed. Therefore, the kinetic energies of photoelectrons excited from the Sn 4d and Zn 3p core levels, which we use for quantifying X_PES_, lie in a range where the IMFP increases with excitation energy, i.e., 220 eV is the most and 4000 eV is the least surface-sensitive measurement.^31^

In order to calculate the IMFP for the Sn 4d- and the Zn 3p-derived photoelectrons for different excitation energies, we used the software SESSA v2.2 and therein the TPP-2M formula,^32^ assuming a material density of 5.8 g/cm^3^ (average value for 20% SnO_2_ and 80% ZnO). Details on the measurement geometry are given in the Supporting Information (Figure S1). Table 2 states the derived average of the IMFP values of both Sn 4d and the Zn 3p core levels for the different excitation energies.

**Table 2:** Average IMFP for the Sn 4d and Zn 3p photoelectrons and X_PES_ = [Sn]/[Me] ratio derived from XPS and HAXPES data recorded with different excitation energies, *h*ν_exc_ ^a^ ^,^ ^32^

*h*ν_exc_	IMFP (nm)	X_PES_		
220 eV	0.59 ± 0.02	0.27 ± 0.02	0.27 ± 0.02	0.30 ± 0.02
1400 eV	2.3 ± 0.1	0.18 ± 0.02	0.18 ± 0.02	0.24 ± 0.02
4000 eV	5.4 ± 0.3	0.12 ± 0.02	0.13 ± 0.02	0.17 ± 0.02
from Table 1	X_XRF_	0.188	0.210	0.252

a For comparison, the XRF-derived composition is also depicted.

### Quantitative Analysis of the X-ray Photoelectron Spectroscopy Data

The elemental composition of the R200 ZTO layers as well as the interlayers was derived by determining the peak intensities (i.e., peak areas) extracted from detail spectra of Zn 3p, Sn 4d, and Ga 2p_3/2_ photoemission lines using the software fityk.^33^ For all peaks, we used Voigt profile functions in combination with linear backgrounds. Thereby, for spin–orbit doublets, a pair of Voigt functions with the same peak width and intensity ratios obeying the spin 2j+1 multiplicity rule was employed. Due to the overlap with O 2s, the spin–orbit splitting between Sn 4d_3/2_ and Sn 4d_5/2_ was fixed to the literature value of 1.08 eV.^34–36^ The peak intensities extracted from the fit were then corrected for the respective IMFP,^32^ the photoionization cross section,^32,37,38^ and the transmission function of the analyzer.^39^ The derived IMFP-dependent composition is given in Table 2.

Using SESSA v2.2, the Ga_2_O_3_/ZnO ratio (*z*) of the (Ga_2_O_3_)_*z*_ZnO interlayer was estimated by simulating the Ga–O and Ga–Se contributions to the Ga 2p_3/2_ line in model systems with different z consisting of an infinitely thick ACGSe absorber, a 3 nm thick interlayer of (Ga_2_O_3_)_*z*_ZnO (more details about the interlayer later in the manuscript), and a 12.5 nm thick ZTO layer whose composition was determined by XPS measurements using a photon energy of 220 eV. A sketch of the model is provided in the inset of Figure S1. The densities used for the simulations were the composition-weighted averages of the densities of ZnO and Ga_2_O_3_ for the interlayer and ZnO and SnO_2_ for the ZTO.

## Results and Discussion

First, we will discuss the device performance of the different ZTO/ACGSe-based solar cells documented by the current–voltage (*J*(*V*)) and external quantum efficiency (EQE) measurements shown in Figure 1 and reported in ref 19. Table 3 compares the *J*(*V*) parameters of the best cells containing ZTO buffers with different X_XRF_ = [Sn]/[Me] values, grown via the R200 ALD process, to the best cell with X_XRF_ = 0.260, grown in the F120 reactor, as published in an earlier study.^19^ The direct comparison of the two samples with similar X_XRF_ in the buffer (X_XRF_ = 0.252 vs 0.260) produced in ALD reactor R200 and F120, respectively, reveals several differences: The open-circuit voltage (*V*_OC_) is slightly lower for the device with the R200-processed ALD layer, while the short-circuit current density (*J*_SC_) value is rather unchanged, as corroborated by the EQE in Figure 1b. However, the most remarkable difference is a massive reduction in the FF caused by a kink in the *J*(*V*) characteristic (see Figure 1a, leading to a 2.8% lower efficiency (8.4% vs 11.2%, see Table 3).

**Table 3:** *J*(*V*) parameters of the best ZTO/ACGSe-based cells with the (Zn_1–*x*_Sn_*x*_)­O_*y*_ layers prepared in the R200 or F120 ALD-reactor with X_XRF_ = [Sn­[/[Me] ranging between 0.188 and 0.260 (cell performance for the device based on the F120-processed ZTO from ref 19)­^a^

ALD process	X_XRF_	*FF* (%)	*V*_OC_ (mV)	*J*_*SC*_ (mA/cm^2^)	η (%)
R200	0.188	56.6	857	18.8	9.1
R200	0.200	54.7	849	18.8	8.7
R200	0.252	46.8	950	18.9	8.4
F120	0.260 ^19^	61.0	985	18.6	11.2

a Performance is shown after 24 h of light soaking; no anti-reflection coating was used.

In order to shed light on the underlying mechanism that might explain the performance gap, we looked at the chemical interface structure. Figure 2 shows dark-field STEM cross-section images of full devices (i.e., including ZnO:Al top electrode) based on the ZTO/ACGSe stacks with R200- and F120-processed ZTO with similar compositions (i.e., *X*_XRF_ = 0.252 and 0.260, respectively) and corresponding EDS line scans. While the image of the R200-processed ZTO-based stack (Figure 2a) exhibits a dark line between the ACGSe absorber and the ZTO buffer (which strongly suggests the presence of an interlayer of only a few nanometers thickness that formed between absorber and buffer), this dark line is missing in the image of the F120-processed stack (Figure 2b, suggesting the formation of a rather direct interface between the absorber and buffer without any sign of interlayer formation). Note that the dark color in the dark-field STEM image indicates that fewer electrons are scattered in the interlayer than in its surroundings, suggesting, for example, the presence of an amorphous material.

Figure 2c shows the EDS line scan of the solar cell with R200-processed ZTO, with the position and scanning direction indicated by the arrow in Figure 2a. The normalized atomic concentrations of the different elements are plotted as a function of distance to the buffer–absorber interface at around 0 nm. The Zn and O signals rise about 3 nm before the Sn signal. Further, the Sn signal indicating the width of the actual ZTO layer is 12.5 nm wide (note that the layer thickness is about 9 nm thinner than what was measured on the glass substrate by XRF (see Table 1, last column). This discrepancy can be explained by the fact that the initial ALD growth is not inhibited on glass, unlike on chalcopyrite materials (see discussion above). In addition, the Ga signal of the absorber does not go to zero at about 5 nm like the Se signal but penetrates further into the buffer layer (note that the nonzero Ag signal in the buffer and the ZnO window is an artifact arising from the peak overlap of the Ag L- and Ar K-line. The Ar stems from the Ar plasma used for sputtering. In addition, the noise of the Ag signal outside the absorber layer appears larger after normalization since it has the smallest concentration of all ACGS elements. The nonzero Cu signal outside the absorber region arises from the used Cu TEM grid.) These observations corroborate that an about 3 nm thick interlayer is formed, consisting predominantly of a mixture of gallium, oxygen, and zinc atoms. Further, the homogeneously textured contrast of the ZTO layer in the dark-field STEM image (Figure 2a) as well as the shape of the Sn- and the O-signal in the line scan (Figure 2c) do not imply a significant composition gradient within ZTO.

In contrast, the EDS line scan of the sample with the F120-processed ZTO shown in Figure 2d demonstrates that the Sn signal rises parallel to the O and the Zn signal in the interface region, agreeing with the STEM image-based conclusion of the presence of a rather abrupt ZTO/ACGSe interface in this case. This discrepancy between the layer stacks prepared in the R200 and in the F120 reactors is further supported by EDS maps shown in Figure S2 in the Supporting Information.

In order to get further insights into the chemical structure of the ZTO buffer and the interlayer formed between R200-processed ZTO and the ACGSe absorber, photon-energy and thus depth-dependent X-ray photoelectron spectroscopy measurements were performed using soft and hard X-rays (i.e., 220, 1400, and 4000 eV). Figure S3 shows 4000 eV excited HAXPES survey spectra of the three R200-processed ZTO/ACGSe sample stacks with different X_XRF_ values, together with that of bare ACGSe. The bare absorber spectrum exhibits peaks related to the absorber elements (as expected), as well as to Na, which has diffused to the absorber surface from the NaF precursor layer. In addition, small peaks arising from oxygen and carbon can be observed, which we ascribe to minor surface contamination. The ZTO/ACGSe spectra are dominated by intense peaks associated with the buffer elements Sn, Zn, and O. Close inspection of the HAXPES data (insets in Figures S3 and S4) reveals the presence of small peaks related to Ga 2p_3/2_ and Se 3p as well as from Cu 2p and Ag 3d. This suggests that either the buffer layers are not fully closed or the photoelectrons from the respective core levels have enough kinetic energy to pass the entire buffer layer without being inelastically scattered. Since more surface-sensitive XPS spectra of the shallow core levels recorded with 220 eV (IMFP <1 nm) do not display peaks associated with absorber elements (see Figure S5), we conclude the layers are closed, and the information depth of the 4000 eV excited HAXPES measurements is sufficient to monitor the entire buffer layer including the interface to the ACGSe.

For investigating the chemical states of Sn and Zn in the buffer layers and revealing whether they change within the ZTO layer or not, we focus on the Sn 4d and the Zn 3p peaks, as these can be excited with all the photon energies used and are, unlike, e.g., Zn 3d, not strongly influenced by the valence band. Figure 3 exhibits detail spectra of both core levels measured with the different excitation energies for the R200-processed ZTO/ACGSe sample with an X_XRF_ of 0.252. For the ZTO/ACGSe samples with different compositions (X_XRF_ = 0.188 and 0.210), the spectra are displayed in Figures S6 and S7. Both Sn 4d and Zn 3p can be fitted with one doublet whose binding energy (BE) is consistent with literature values for SnO_*x*_ and ZnO, respectively, as the comparison in Table 4 shows. Therefore, we conclude that there is no significant contribution of secondary phases such as residual ALD precursors.

**Figure 3:**
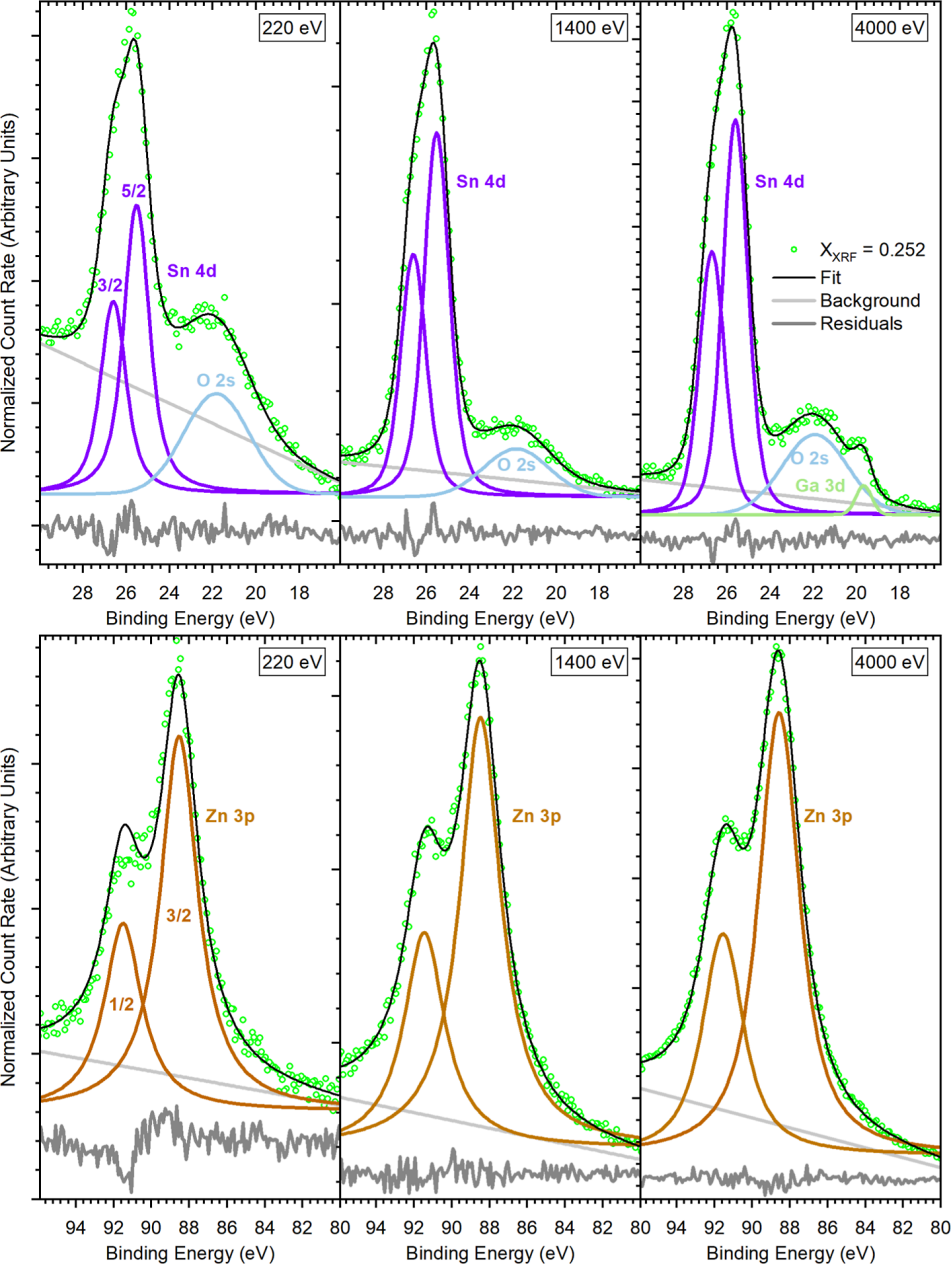
220 eV (left), 1400 eV (center), and 4000 eV (right) excited XPS/HAXPES detail spectra (including fit) of the Sn 4d (top) and the Zn 3p (bottom) photoemission line for the R200-processed ZTO/ACGSe sample with X_XRF_ = 0.252. Note that the Sn 4d spectra partially overlap with the O 2s line, and (for the most bulk sensitive measurement) also, the Ga 3d signal from the ACGSe absorber has to be considered in the fit analysis. The used linear background and residuum (difference between fit and data) are also shown.

**Table 4:** Binding energies (BE) in eV extracted from the fits of the XPS/HAXPES data of the ZTO/ACGSe samples with (Zn_1–*x*_Sn_*x*_)­O_*y*_ layers prepared in the R200 ALD reactor with X_XRF_ values ranging between 0.188 and 0.252 shown in Figures 3, S6, and S7, together with literature values of metallic and oxidized Sn and Zn for comparison recorded at 1486.6 and 90 eV, respectively

X_XRF_ →	0.188	0.210	0.252	0.188	0.210	0.252
*h*ν_exc_ ↓	BE (Sn 4d5/2) in eV			BE (Zn 3p3/2) in eV		
220 eV	25.3 ± 0.1	25.5 ± 0.1	25.5 ± 0.1	88.4 ± 0.1	88.5 ± 0.1	88.5 ± 0.1
1400 eV	25.5 ± 0.1	25.6 ± 0.1	25.6 ± 0.1	88.5 ± 0.1	88.6 ± 0.1	88.5 ± 0.1
4000 eV	25.6 ± 0.2	25.7 ± 0.2	25.6 ± 0.2	88.6 ± 0.2	88.6 ± 0.2	88.6 ± 0.2
1486.6 eV (literature)	24.0 ± 0.4 (Sn), 25.3 ± 0.4 (SnO), 25.8 ± 0.4 (SnO_2_)^40–42^	88.8 ± 0.3 (Zn), 89.0 ± 0.3, 88.8 ± 0.2 (ZnO)				
90 eV (literature)	24.0 ± 0.1 (Sn), 25.50 ± 0.02 (SnO), 26.22 ± 0.01 (SnO_2_)^43^					

Comparing the fit-derived Sn 4d_5/2_ binding energies for the most surface-sensitive measurements (220 eV) to literature values with comparable IMFP (0.63 nm for 220 eV vs 0.47 nm for 90 eV, see Table 4) suggests that the chemical structure of Sn at the surface of the ZTO layer is best described by Sn^2+^; i.e., we identify the studied ZTO tentatively as a ZnSnO (and not a ZnSnO_3_ or a Zn_2_SnO_4_, in which tin would be in a Sn^4+^ state)-type material. This is further substantiated by the shape of the valence band spectrum shown in Figure S8. The additional density of states above 2.5 eV (i.e., closer to *E*_F_) may indicate the presence of occupied Sn 5s states, which only occur for Sn^2+^ but not for Sn^4+^ (but could also be attributed to defect states).

To determine the ZTO composition, the peak intensities of Zn 3p_3/2_ and Sn 4d_5/2_ extracted from the fits in Figures 3, S6, and S7 were used and corrected for the respective inelastic mean free path (Table 2), the photoionization cross section,^32,37,38^ and the transmission function of the analyzer.^39^ From these numbers, we calculated the X_PES_ ratio as a function of IMFP. The results are shown in Figure 4 (and Table 2), directly compared to the XRF-derived compositions. The depth-dependent XPS/HAXPES-derived composition profiles for the ZTO samples with X_XRF_ = 0.188 and 0.210 (black and red) agree within the experimental error margin. For all samples, X_PES_ decreases as the measurement becomes more bulk-sensitive. For the most surface-sensitive measurements (*h*ν_exc_ = 220 eV, IMFP = 0.59 ± 0.02 nm, see Table 2) of all samples, X_PES_ is significantly higher than X_XRF_. The probing depth of XRF is such that it gives practically an average composition across the thickness of the film without significant sensitivity gradients due to signal attenuation. The information provided by photoemission measurements, even when using higher photon energy excitation, however, is strongly (exponentially) weighted in favor of the sample surface. The discrepancy between the averaged XRF-derived composition and the XPS/HAXPES-derived composition profile can be explained by three scenarios: (1) There is a composition profile throughout the ZTO layer. (2) A rather Sn-poor interlayer forms toward the absorber interface, on top of which a Sn-rich layer grows. (3) A combination of (1) and (2).

**Figure 4:**
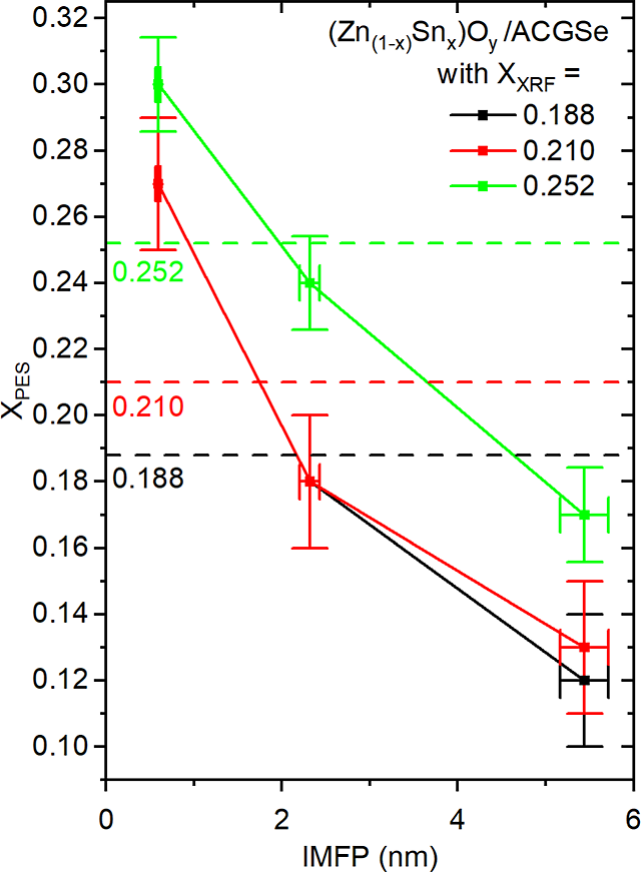
X_PES_ profiles of the studied ZTO/ACGSe layer stacks with X_XRF_ values (for the (Zn_1–*x*_Sn_*x*_)­O_*y*_ buffer) of 0.188, 0.210, and 0.252. This average composition is also indicated by the horizontal dashed lines.

Consulting the STEM and EDS results depicted in Figure 2 and discussed above (briefly: an interlayer is formed consisting predominantly of a mixture of gallium, oxygen, and zinc atoms as well as no indication for a significant composition gradient within ZTO is found), we conclude that scenario (2) most likely applies in the studied case here.

Regarding the chemical composition of the interlayer, the elements found in the line scan suggest that either zinc gallate (ZnGa_2_O_4_) or a mixture of not necessarily stoichiometric ZnO and Ga_2_O_3_ is formed ((Ga_2_O_3_)_*z*_ZnO). For the former, a significant shift of the Ga 2p_3/2_ line by about 0.6 eV to higher binding energies with respect to that of Ga_2_O_3_ is expected.^44^ In Figure S9a–d, the Ga 2p_3/2_ region of the bare ACGSe as well as the ZTO/ACGSe layer stacks measured with an excitation energy of 4000 eV are displayed. All spectra can be fitted with two Voigt functions, a main one (BE = 1117.65 ± 0.06 eV) which we assign to Ga–Se bonds in the absorber material and a secondary one (BE = 1118.39 ± 0.05 eV) which we attribute to Ga–O bonds in the interlayer or, in the case of the bare ACGSe, to Ga–O bonds formed as a result of surface oxidation. Simulating the ratio of Ga–O to Ga–Se bonds for a (Ga_2_O_3_)_*z*_ZnO/ACGSe layer stack with SESSA v2.2 yields a submonolayer surface coverage for the bare absorber. This may act as the foundation of the observed interlayer. As a comparison between the peak positions of the oxide-related feature on the bare ACGSe and the layer stacks shows, there is no significant shift toward higher binding energies when the ZTO is added. Therefore, we conclude that no ZnGa_2_O_4_ is formed, and the interlayer is best described by a mixture of ZnO and Ga_2_O_3_.

Next, we attempt to estimate the (average) ratio of Ga_2_O_3_/ZnO (z) in the (Ga_2_O_3_)_*z*_ZnO interlayer. Note that for the presumably amorphous interlayer (see discussion in conjunction with the TEM data depicted in Figure 1a), a wide range of compositions can be expected. Unfortunately, a differentiation between the Zn 3p signal arising from the interlayer and that from the ZTO buffer layer is not possible, as the chemical state of Zn is the same in both layers, namely, Zn^2+^. Hence, we simulate the Ga 2p_3/2_ intensities of the layered ZTO/(Ga_2_O_3_)_*z*_ZnO/ACGSe system as described in the Methods section to estimate the Ga_2_O_3_ content in the interlayer. The results of the simulations are displayed in Figure S9e together with the experimental results. This approach suggests *z* in the interlayer to be on the order of 1%, which is in seeming contradiction to the Ga_2_O_3_ interlayer content suggested by the pronounced Ga shoulder in the EDS line scan in Figure 2c. However, a robust quantification of the Ga concentration in the interlayer is not possible due to the very small extension of only 3 nm, the lateral resolution of the STEM-EDS technique, and the potentially tilted interface along the depth of the ≈80 nm-thin TEM lamella. Therefore, the XPS simulations seem more reliable. Nevertheless, relying on literature values for the density of the interlayer and assuming an equal distribution of Ga_2_O_3_ tentatively leads to an underestimation of the Ga_2_O_3_ content of the interlayer, as discussed in the Supporting Information. In summary, we suggest that z is well below 10%.

The formation of such a (Ga_2_O_3_)_*z*_ZnO interlayer between a chalcopyrite absorber and an ALD-deposited ZTO buffer was to our knowledge not reported so far.^26,45^ Since it is not found in the sample prepared in the F120 ALD reactor, it appears that the ALD process in the R200 reactor is responsible for the growth of the (Ga_2_O_3_)_*z*_ZnO interlayer at the buffer/absorber interface. Several factors associated with the different reactor designs may contribute to this. First, the reactor volume of the F120 reactor is much smaller (∼1 cm^3^) than that of the R200 reactor (∼1000 cm^3^). Moreover, the geometry of F120 confines the precursor flows to a narrow channel just above the substrates. Together, this will enhance the effective exposure to the precursors, with much higher local concentrations seen by the substrate in the F120 reactor as compared with in the R200 reactor. We speculate that this is the main reason for the necessity to use a higher Zn/Sn pulse ratio in the R200 reactor to achieve similar [Sn]/[Me] compositions. Second, the larger volume and open design of R200 require longer purging times, making the ALD process slower. Third, indirect heating of the substrate holder makes the warming time and temperature stabilization about three times longer in the R200 reactor. Together, this leads to a slower ALD process and higher temperature budget in the R200 reactor, which will enhance diffusion of species across, to, and from the growth surface. For example, Ga may diffuse out of the ACGSe, or Zn may diffuse into the ACGSe, leading to a mixed interlayer formation. Based on the relatively low Ga concentration and tailing profile observed, however, it seems more feasible that Ga diffuses into the growing film during the initial ALD stages. Another possibility is that Ga already present at the ACGSe surface is oxidized during the initial stages of ALD, or even during the relatively long warm-up time due to sufficient (trace) amounts of water and/or oxygen in the carrier gas. In this case, a more distinct Ga containing oxide layer could be expected to form at the interface, but roughness and limited depth resolution make it hard to distinguish between these scenarios and related possibilities without a more careful investigation. Potential mitigation strategies could include the use of a load-lock system as it would substantially reduce the required temperature budget, or the addition of an atomic layer etching step^46^ in the process to remove Ga_2_O_3_ from the ACGSe surface prior to ZTO growth.

Finally, we discuss the impact of the (Ga_2_O_3_)_*z*_ZnO interlayer on the solar cell performance. Table 3 compares the PV parameters of the ZTO/ACGSe-based devices with (R200-processed) and without (F120-processed) the interlayer. The most remarkable difference is a massive reduction in *FF*, leading to a 2.8%-point lower efficiency (8.4% vs 11.2%) for *x* = 0.25–0.26, strongly suggesting a transport barrier for electrons created by the interlayer. To explain this finding, we take into consideration our estimation that the Ga_2_O_3_/ZnO ratio in the interlayer is well below 10%. According to a study by Chen and co-workers, below a Ga_2_O_3_ content of around 11%, corresponding ZnGaO thin films have similar band onset positions and energy band gaps to ZnO.^47^ Therefore, we suggest in a zeroth-order approximation that the interlayer has an electronic structure similar to that of pure ZnO, i.e., having a strong n-type character with the Fermi level (*E*_F_) being close to the conduction band minimum (CBM). The formation of a cliff-like CB offset with the p-type ACGSe absorber (i.e., the CBM of the absorber is located above the CBM of the interlayer) is thus a reasonable conclusion. Based on the suggestion^45^ that the CBM of the ZTO buffer is also located above that of ZnO (which we found to be electronically similar to the formed (Ga_2_O_3_)_*z*_ZnO interlayer), we speculate that the CB alignment across the ZTO/(Ga_2_O_3_)_*z*_ZnO/ACGSe heterostructure may form an electron trap, i.e., compared to the low CBM of the (Ga_2_O_3_)_*z*_ZnO interlayer—high CBM of the ZTO and ACGSe form a “well” trapping the photogenerated electrons. This is corroborated by the observation that among the solar cells with a R200-processed ZTO buffer layer, the FF value deteriorates with increasing Sn content (see Table 3), indicating that in the above outlined situation, the CBM of the ZTO moves away from *E*_F_ with increasing Sn content, and hence, the CBM difference between the interlayer and ZTO, and thus the electron transport barrier, rises.

Overall, this work shows how varying ALD-ZTO process conditions, leading to similar integral and average compositions, can result in very different growth dynamics, potentially creating an interlayer that is detrimental to solar cell performance. Considering the increasing popularity of ZTO as a low-affinity buffer that allows for higher *V*_OC_ values in different thin-film PV technologies,^3,48–52^ such knowledge is of great value for the research community.

## Conclusions

This study investigates the impact of the atomic layer deposition settings on the chemical structure of the (Zn_1–*x*_Sn_*x*_)­O_*y*_/wide-gap (Ag,Cu)­GaSe_2_ thin-film solar cell interface using transmission electron microscopy and X-ray spectroscopy techniques. When growing the ZTO in an industrial-scale reactor instead of in a lab-scale reactor, we find that ZTO no longer grows directly on the absorber material but forms an ∼3 nm thick interlayer best described by a mixture of ZnO and Ga_2_O_3_. Using simulations of photoelectron spectra, we found the Ga_2_O_3_ content to be well below 10%. Since there is no evidence of such an interlayer in similar layer stacks prepared in a lab-scale ALD reactor, we identify differences in the reactor design, especially the larger volume, to slow down the ALD process and increase the temperature budget, enhancing diffusion of species across, to, and from the growth surface, e.g., Ga from the ACGSe toward the ZTO growth surface, thus forming the interlayer. Alternatively, and/or additionally, the longer warm-up and process time may foster the formation of surface GaO_*x*_ prior to or in the first stages of ZTO formation that may act as the foundation for the interlayer.

Further, the interlayer may likely be the reason for a significant fill factor and efficiency loss in the respective solar cell devices. Assuming that the electronic structure of the interlayer is very similar to ZnO, we attribute the fill factor loss to its high electron affinity, resulting in an electron trap at the ZTO/(Ga_2_O_3_)_*z*_ZnO/ACGSe heterostructure. In summary, our results underline the importance of monitoring the results of ALD processes and are particularly important to consider when changing from small, lab-scale reactors to industrial-scale setups.

## Supplementary Material



## Notes

The Supporting Information is available free of charge at https://pubs.acs.org/doi/10.1021/acsami.5c21162. The raw data has been deposited online in a Zenodo repository and has the following permanent DOI: 10.5281/zenodo.17406468.

Geometric parameters of the SISSY I endstation and sketch of the model used for SESSA simulations, additional dark-field STEM images and EDS maps of ACGSe-based solar cells prepared with ZTO buffer layers prepared in F120 and R200 reactors HAXPES survey spectra of R200-processed buffer/absorber stacks with different (Zn_1‑x_Sn_*x*_)­O_*y*_ compositions and a bare ACGSe absorber sample, HAXPES spectra of Cu 2p and Ag 3d photoemission lines of R200-processed buffer/absorber stacks with different (Zn_1‑x_Sn_*x*_)­O_*y*_ compositions and of an ACGSe bare absorber sample, 220 eV excited XPS spectra of the shallow core-level region of all R200-processed buffer/absorber stacks with different (Zn_1‑x_Sn_*x*_)­O_*y*_ compositions and of a bare ACGSe absorber sample, XPS/HAXPES detail spectra of the Sn 4d and the Zn 3p photoemission line for the R200-processed ZTO/ACGSe sample with X_XRF_ = 0.188 and 0.210 composition including fit 220 eV excited detail spectra of the valence band regions measured for all R200-processed buffer/absorber samples with different (Zn_1–*x*_Sn_*x*_)­O_*y*_ compositions, HAXPES detail spectra of the Ga 2p_3/2_ region of all R200-processed buffer/absorber stacks with different (Zn_1–*x*_Sn_*x*_)­O_*y*_ compositions and of a bare ACGSe absorber sample including fit, and comparison of I_Ga–O_/I_Ga–Se_ ratios measured on all R200-processed buffer/absorber stacks with different (Zn_1–*x*_Sn_*x*_)­O_*y*_ compositions and of a bare ACGSe absorber sample to *I*_Ga–O_/*I*_Ga–Se_ derived from simulated spectra of layered ZTO/(Ga_2_O_3_)_*z*_ZnO/ACGSe systems for different values of *z* = Ga_2_O_3_/ZnO (PDF)
