# Supplementary material for: How ALD Settings May Affect the Chemical Structure of the (Zn1–x Sn x )O y /Wide-Gap (Ag,Cu)GaSe2 Thin-Film Solar Cell Interface
Source: ACS Appl Mater Interfaces. 2026 Jan 16;18(3):6180–90. doi: 10.1021/acsami.5c21162 (PMC12862760; doi:10.1021/acsami.5c21162)
Supplement: Supplementary file 1 [file am5c21162_si_001.pdf]

Supporting Information

How ALD settings may affect the chemical structure of the $(Zn_{1-x}Sn_x)O_y$/wide-gap $(Ag,Cu)GaSe_2$ thin-film solar cell interface

Angelika Demling[1], Jan Keller[2], Regan G. Wilks[1,3], Carl Hägglund[2], Marika Edoff[2], Marcus Bär[1,3,4,5]*

[1]Department of Interface Design, Helmholtz-Zentrum Berlin für Materialien und Energie GmbH (HZB), 12489 Berlin, Germany, email: angelika.demling@helmholtz-berlin.de

[2]Division of Solar Cell Technology, Department of Materials Science and Engineering, Uppsala University, P. O. Box 35-751 03, Uppsala, Sweden

[3]Energy Materials In-Situ Laboratory Berlin (EMIL), HZB, 12489 Berlin, Germany

[4]Department of X-ray Spectroscopy at Interfaces of Thin Films, Helmholtz-Institute Erlangen-Nürnberg for Renewable Energy (HI ERN), 12489 Berlin, Germany

[5]Department of Chemistry and Pharmacy, Friedrich-Alexander-Universität Erlangen-Nürnberg (FAU), 91058 Erlangen, Germany

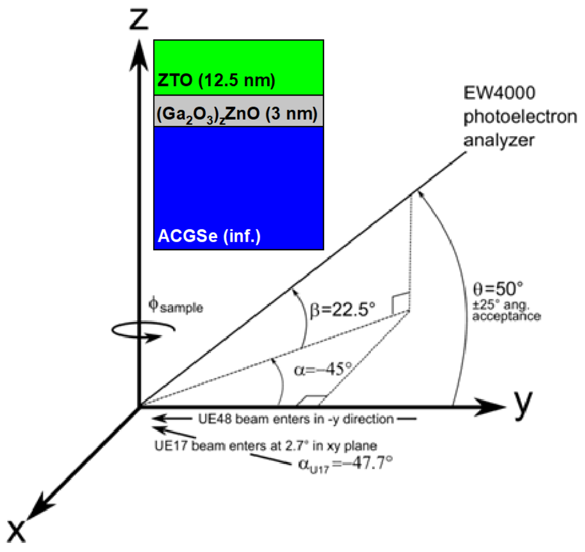

Figure S1: Important geometric parameters of the SISSY-I endstation. Axis labels x-y-z are based on internal coordinate system of 5-axis manipulator. z axis is vertical. Inset: Sketch of model used for SESSA simulations aiming at deriving z = Ga_2O_3/ZnO.

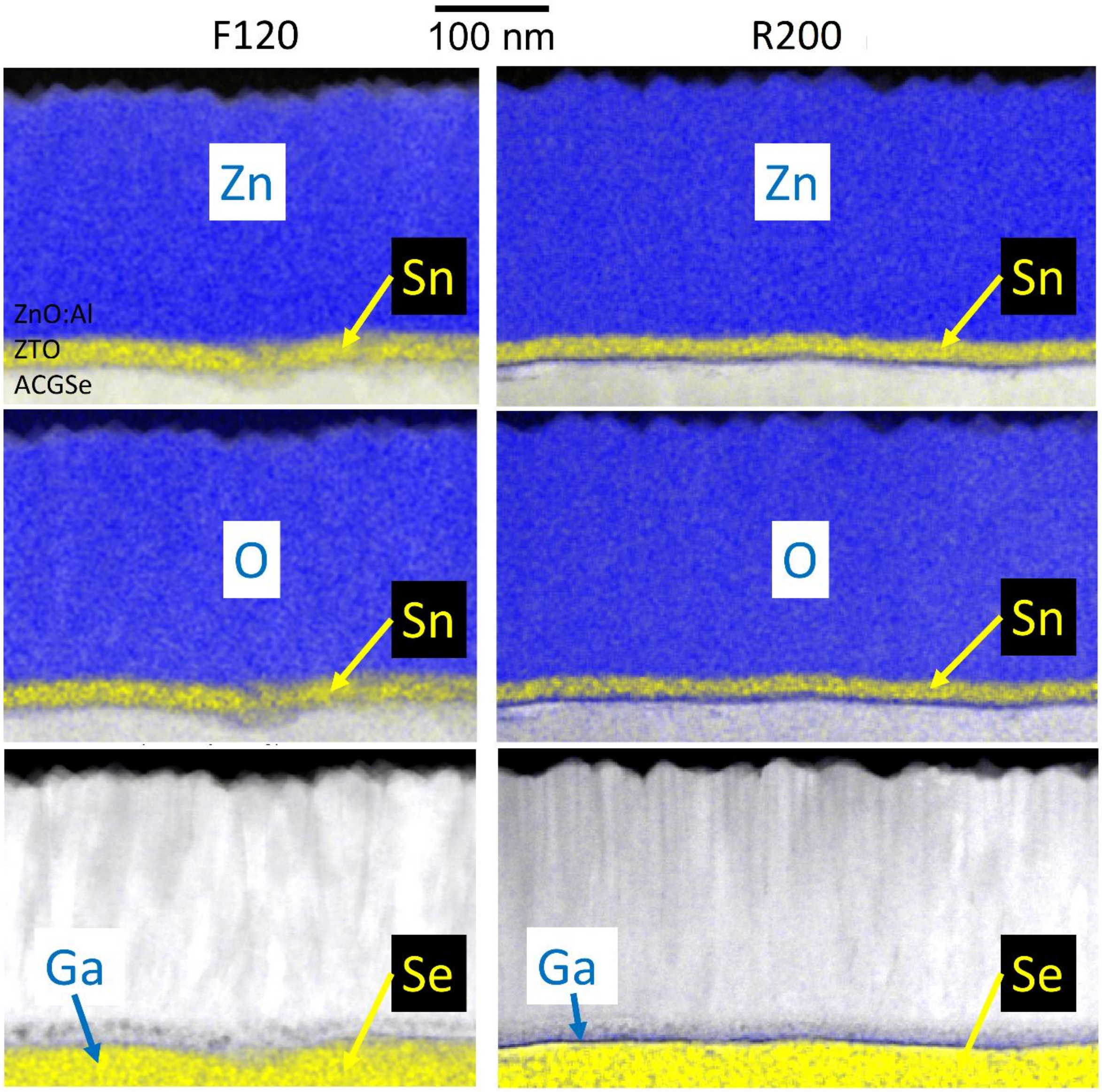

Figure S2: Overlays of the dark field STEM images and EDS maps of ACGSe based solar cells prepared with ZTO ($(Zn_{1-x}Sn_x)O_y$) buffer layers prepared in an F120 (left, $X_{XRF} = 0.26$) and a R200 reactor (right, $X_{XRF} = 0.252$). The pixel opacity of the elemental maps is set linear to the respective normalized atomic concentrations.

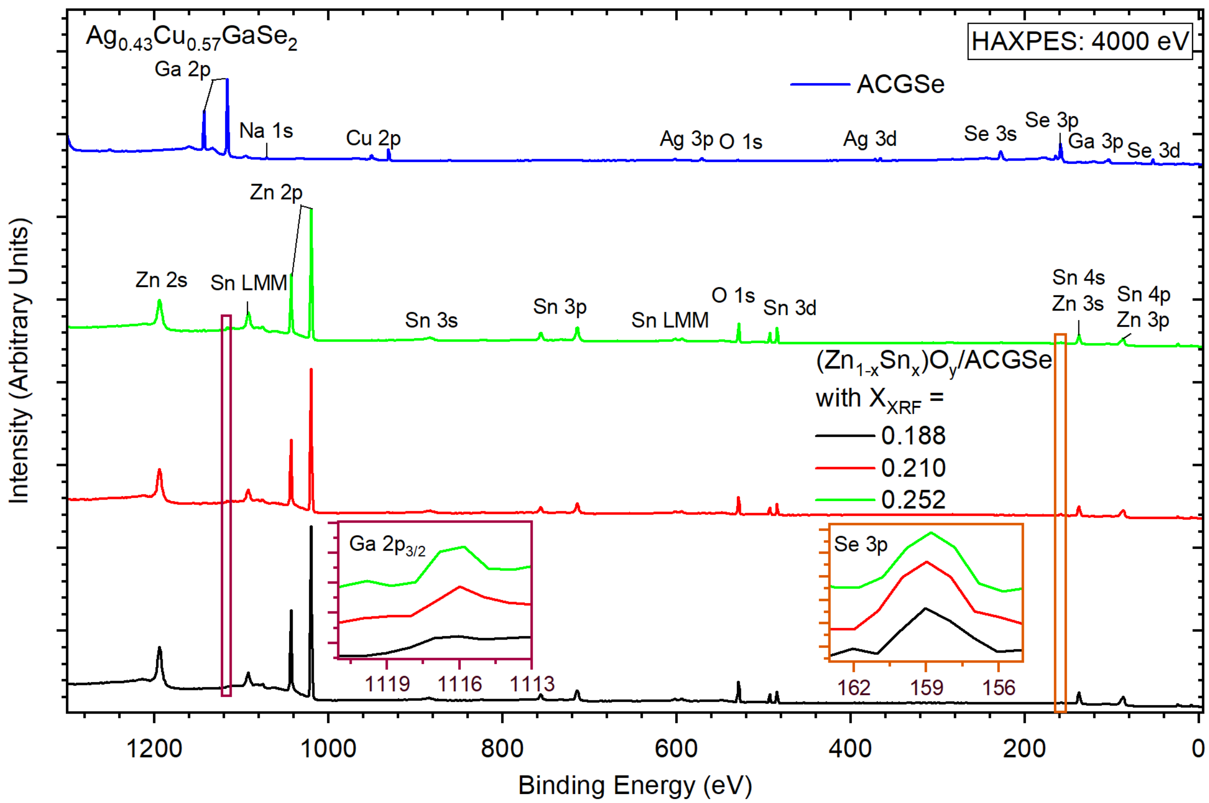

Figure S3: HAXPES survey spectra excited with 4000 eV of R200 processed buffer/absorber stacks (bottom three spectra) with different $(Zn_{1-x}Sn_x)O_y$ compositions and a bare ACGSe absorber sample (top spectrum) for comparison. The insets show enlarged images of the Ga $2p_{3/2}$ and the Se 3p region of the buffer/absorber stacks, respectively. Vertical offsets were added for clarity.

In order to gain information about the chemical composition of the ZTO layers from the surface to their interface with the ACGSe absorber, HAXPES spectra were acquired using 4000 eV excitation energy (IMFP < 6 nm). Figure S3 shows survey spectra of all three ZTO/ACGSe sample stacks together with that of a bare ACGSe produced in the same absorber deposition process. The bare absorber spectrum exhibits peaks related to the absorber elements (as expected), as well as to Na which has diffused into the absorber (surface) NaF precursor layer. In addition, close inspection of the spectra reveals the presence of small peaks arising from oxygen and carbon, which we

ascribe to minor surface contamination. The ZTO/ACGSe spectra are dominated by intense peaks associated with the buffer elements Sn, Zn, and O. The differences in intensity, particularly that of the Zn 2p peaks, agree at least qualitatively with the Zn content variation among the samples.

As the insets in Figure S3 show, in addition to the peaks related to the buffer material, we find small peaks related to Ga and Se arising from the ACGSe underneath the buffer. Furthermore, features arising from Cu 2p and Ag 3d are detected as well in HAXPES detail spectra as Figure S4 displays. These findings suggest that either the buffer layers are not fully closed, or the photoelectrons from the respective core levels have enough kinetic energy to penetrate the entire buffer layer. However, the former can be excluded as cause of the appearance of Se and Ga peaks in the HAXPES spectra, as more surface-sensitive XPS spectra of the shallow core levels recorded with 220 eV (IMFP < 1 nm) do not display peaks associated with absorber elements (see Figure S5). Hence, we conclude the layers are closed and the information depth of the HAXPES measurement is sufficient to monitor the entire buffer layer including the interface to the ACGSe.

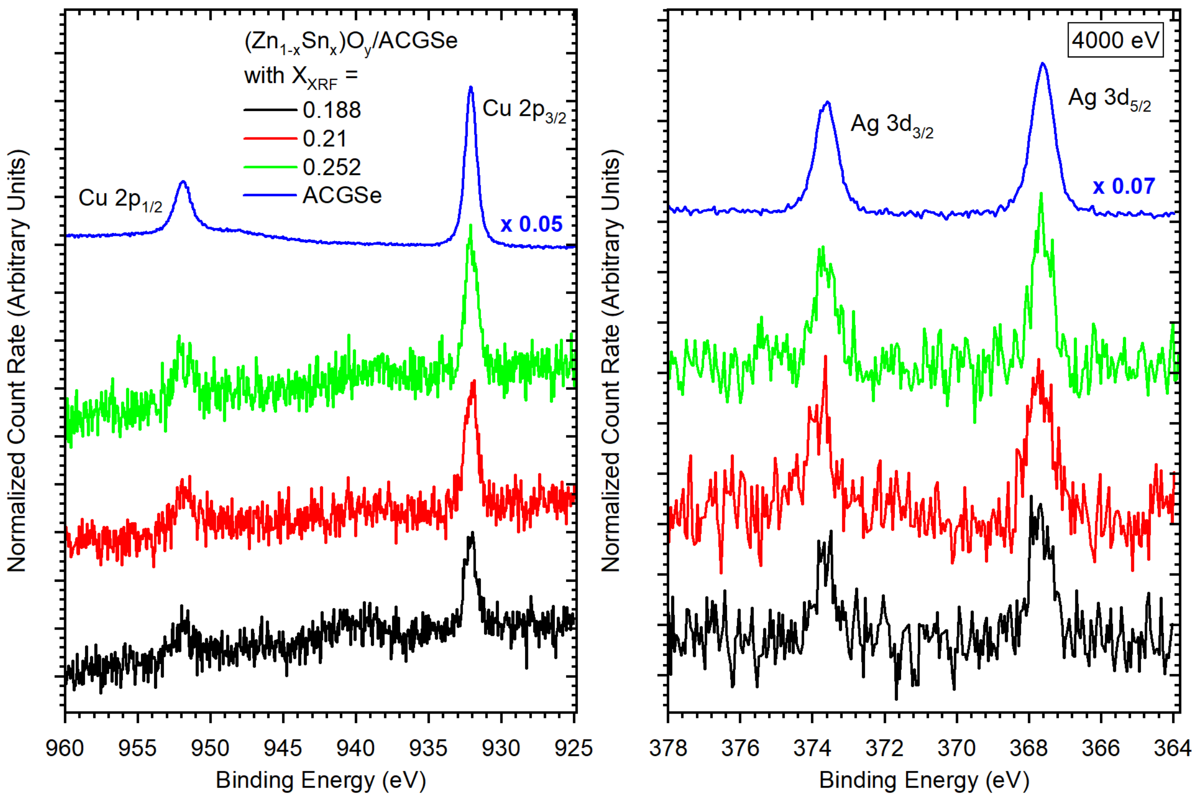

Figure S4: HAXPES spectra of Cu 2p (left) and Ag 3d (right) photoemission lines of R200 processed buffer/absorber stacks (bottom three spectra) with different $(Zn_{1-x}Sn_x)O_y$ compositions and of a ACGSe bare absorber sample (top spectrum, note the magnification factors) for comparison. Vertical offsets were added for clarity.

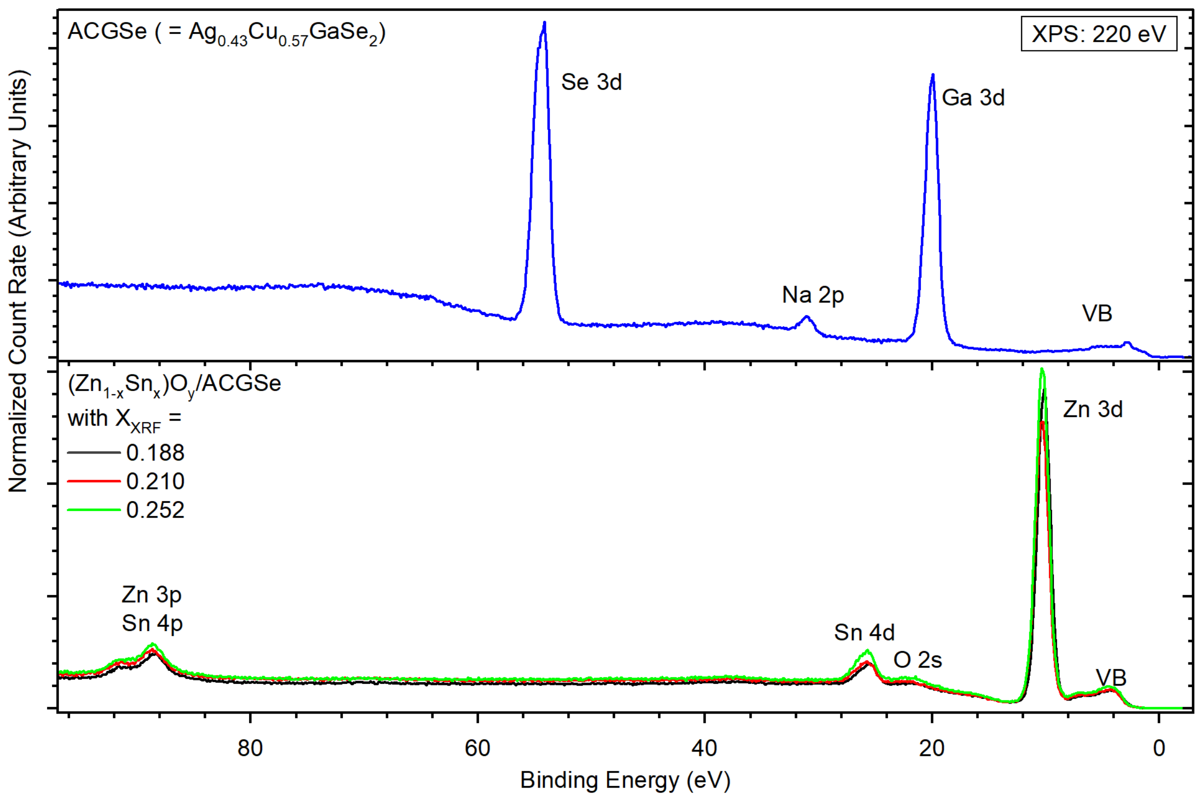

Figure S5: 220 eV excited XPS spectra of the shallow core level region of all buffer/absorber stacks (bottom panel) with different $(Zn_{1-x}Sn_x)O_y$ compositions and of a bare ACGSe absorber sample (top panel) for comparison.

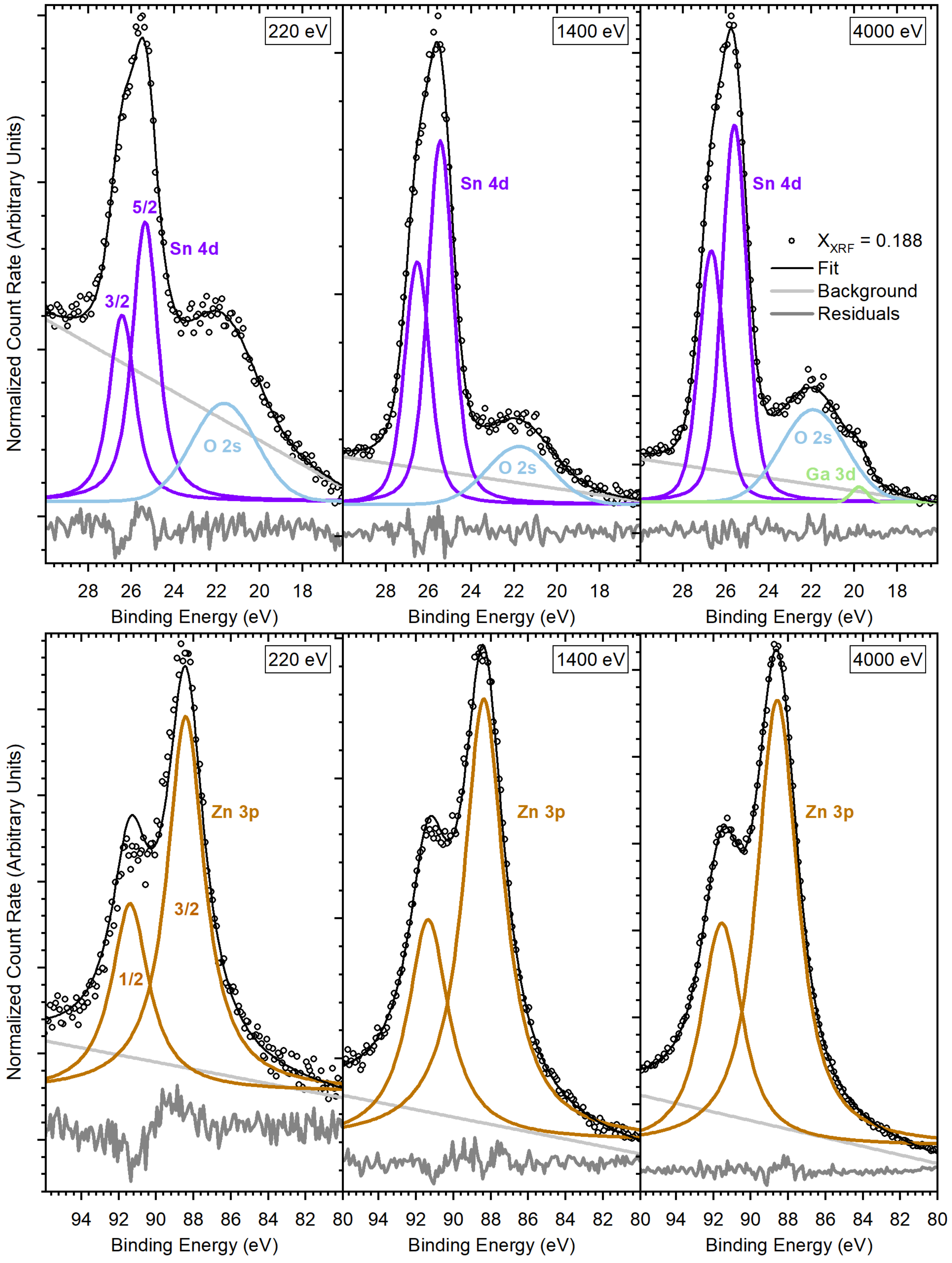
220 eV
1400 eV
4000 eV
5/2
3/2
Sn 4d
O 2s
Ga 3d
X_XRF = 0.188
Fit
Background
Residuals
Zn 3p
1/2
Normalized Count Rate (Arbitrary Units)
Binding Energy (eV)

Figure S6: 220 eV (left), 1400 eV (center), and 4000 eV (right) excited XPS/HAXPES detail spectra (including fit) of the Sn 4d (top) and the Zn 3p (bottom) photoemission line for the R200 processed ZTO/ACGSe sample with a X_{XRF} = 0.188 composition. Note that the Sn 4d spectra partially overlap with the O 2s line and (for the most bulk sensitive measurement) also the Ga 3d signal from the ACGSe absorber must be considered in the fit analysis. The used linear background and residuum (difference between fit and data) is also shown.

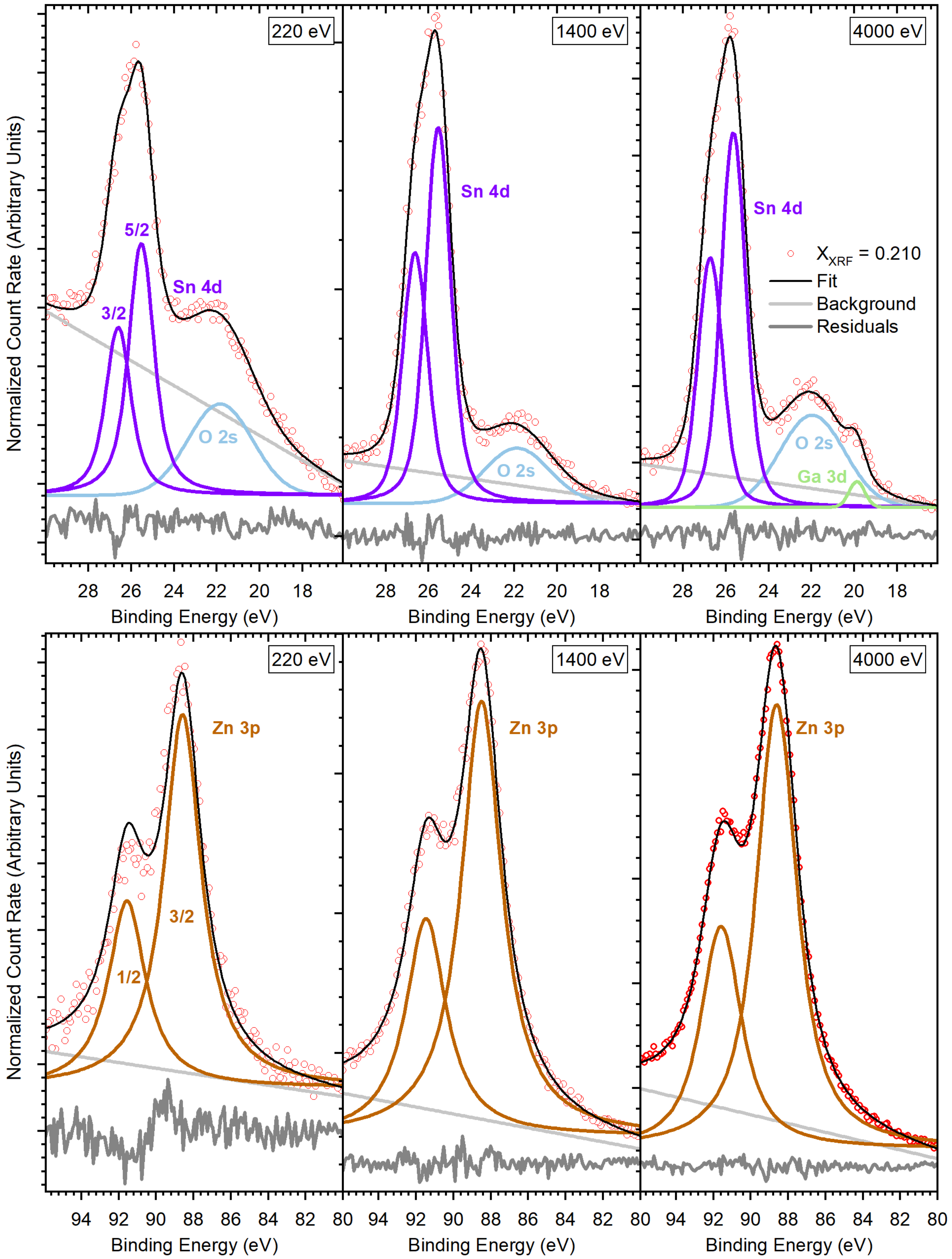
220 eV
1400 eV
4000 eV
Sn 4d
5/2
3/2
O 2s
Ga 3d
X_XRF = 0.210
Fit
Background
Residuals
Normalized Count Rate (Arbitrary Units)
Binding Energy (eV)
28 26 24 22 20 18
Zn 3p
3/2
1/2
94 92 90 88 86 84 82 80

Figure S7: 220 eV (left), 1400 eV (center), and 4000 eV (right) excited XPS/HAXPES detail spectra (including fit) of the Sn 4d (top) and the Zn 3p (bottom) photoemission line for the R200 processed ZTO/ACGSe sample with a X_{XRF} = 0.210 composition. Note that the Sn 4d spectra partially overlap with the O 2s line and (for the most bulk sensitive measurement) also the Ga 3d signal from the ACGSe absorber has to be considered in the fit analysis. The used linear background and residuum (difference between fit and data) is also shown.

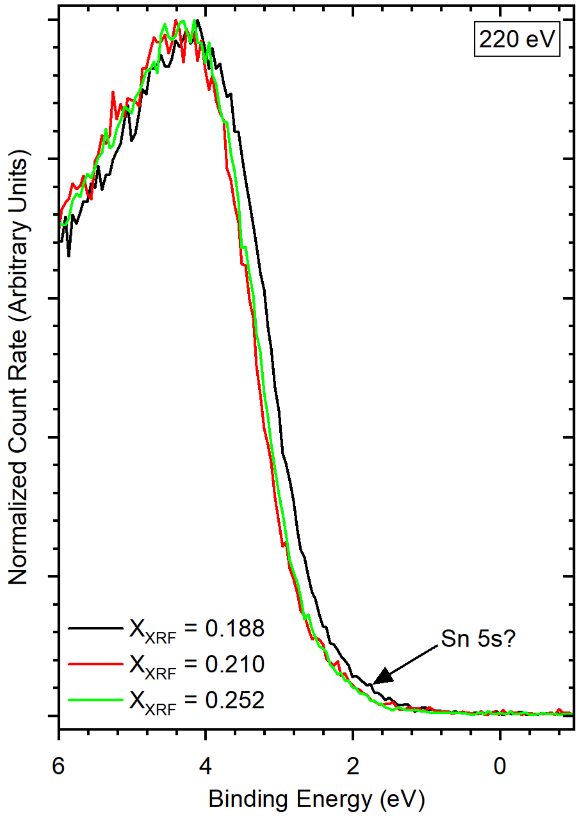

Figure S8: 220 eV excited detail spectra of the valence band regions measured for all R200 processed buffer/absorber samples with different $(Zn_{1-x}Sn_x)O_y$ compositions.

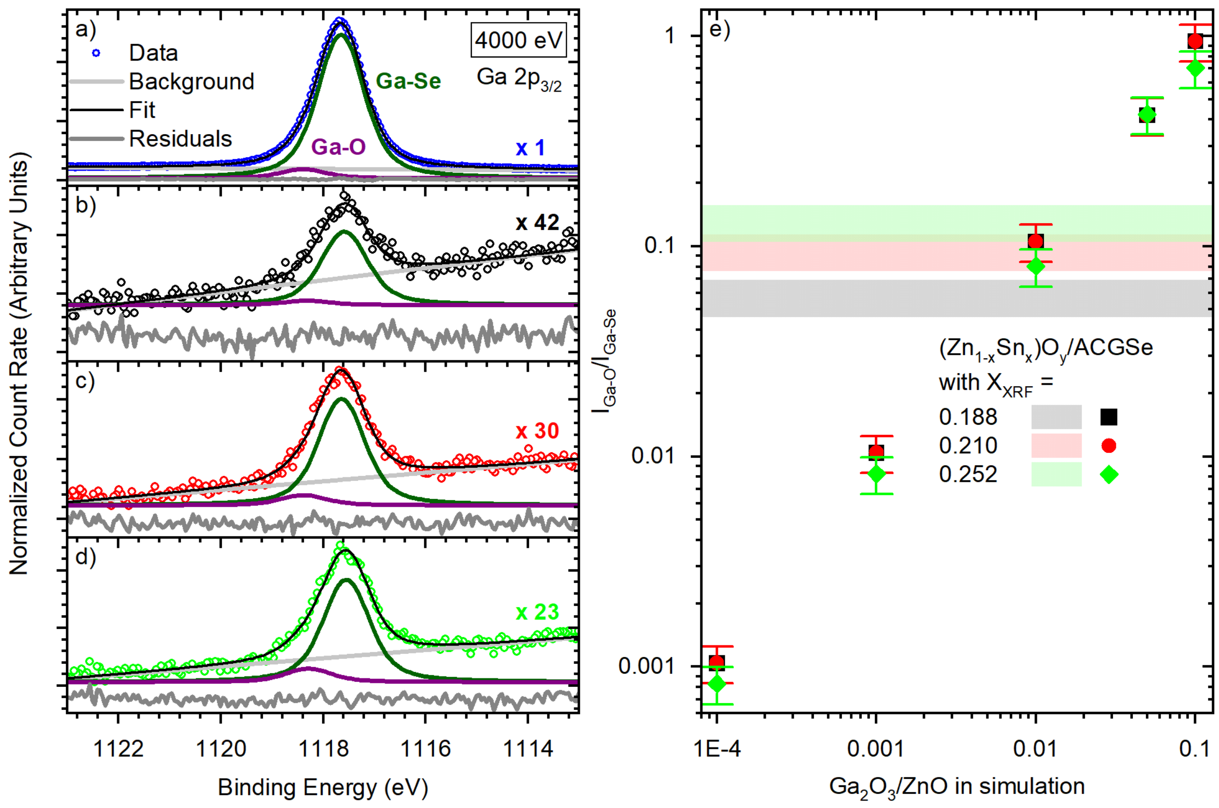

Figure S9: 4000 eV excited HAXPES detail spectra of the Ga $2p_{3/2}$ region measured on a) a bare ACGSe absorber sample, and R200 processed ZTO/ACGSe sample stacks with an average ZTO composition of b) $X_{XRF} = 0.188$, c) $X_{XRF} = 0.210$, d) $X_{XRF} = 0.252$. e) Ratio of the peak intensities arising from Ga-O in the interlayer relative to that arising from Ga-Se in the absorber (I_{Ga-O}/I_{Ga-Se}). The shaded areas refer to the ratios (including experimental uncertainty) derived from the fit of the spectra shown in a)-d). The symbols refer to the intensity ratios (I_{Ga-O}/I_{Ga-Se}) derived from simulated spectra of layered ZTO/$(Ga_2O_3)_z$ZnO/ACGSe systems (see inset in Figure S1) for different values of z = Ga_2O_3/ZnO.

In order to estimate the ratio of Ga_2O_3/ZnO (z) in the $(Ga_2O_3)_z$ZnO interlayer, we simulated the Ga $2p_{3/2}$ intensities of layered ZTO/$(Ga_2O_3)_z$ZnO/ACGSe systems (see inset of Figure S1) as described in the Methods section to calculate the peak intensities arising from Ga-O in the interlayer relative to the intensity arising from Ga-Se in the absorber (I_{Ga-O}/I_{Ga-Se}) for different

values of z. The $I_{Ga\text{-}O}/I_{Ga\text{-}Se}$ ratios derived from simulated spectra are displayed as symbols in Figure S9e and the ratios resulting from the fit of the experimentally derived Ga $2p_{3/2}$ spectra (Figure S9a-d) are shown as shaded areas (to also represent the experimental uncertainty). Consulting Figure S9e, we observe that for z = 0.01 the $I_{Ga\text{-}O}/I_{Ga\text{-}Se}$ ratios derived from simulated and experimentally derived data agree, indicating that for the assumed layered ZTO/$(Ga_2O_3)_zZnO$/ACGSe model system the Ga_2O_3/ZnO ratio in the $(Ga_2O_3)_zZnO$ interlayer is 0.01. Note that this value assumes an equal distribution of Ga_2O_3 throughout the entire interlayer. However, since Ga stems from the absorber, it is likely that its concentration decreases towards the ZTO layer leading to a lower $I_{Ga\text{-}O}$ in the measurement. Additionally, for the density of the interlayer we must rely on literature values for Ga_2O_3 and ZnO which apply to crystalline, stoichiometric materials. However, as discussed in the main text the STEM images strongly suggest an amorphous structure of the interlayer, having a lower density. Hence, simulations tentatively underestimate $I_{Ga\text{-}Se}$. Both effects together might lead to an underestimation of the real Ga_2O_3 content, which is according to test simulations less than an order of magnitude.
